# Report of a Case of Traumatic Empyena

**Published:** 1893-06

**Authors:** N. T. Dulaney

**Affiliations:** Bristol, Tenn.


### REPORT OF A CASE OF TRAUMATIC EMPYEMA.

BY N. T. DULANEY, M. D., BRISTOL, TENN.

Pus being a product of inflammation, its presence implies a present or preceding inflammation, either common or specific, idiopathic or traumatic.

Accumulations of pus in different situations, and under different circumstances, are, by common consent, discussed under different names, thus: "Psoas Abscess," "Lumbar Abscess," and "Palmar Abscess," are terms expressive of certain circumscribed collections of pus, familiar to you all. The term, Hypopyon, is applied to a collection of pus in the anterior chamber of the eye, and to such accumulation in the pleural cavity, the name "Pyothorax" or Empyema is given.

The case which forms the basis of this report is one of extreme interest, from several considerations as will appear as we proceed. Mr. E. G., the subject, is unmarried, aged (30) thirty years, conductor on a freight train on the Norfolk and Western Railroad. Weight, (190) one hundred and ninety pounds.

On August 19th, 1891, in the discharge of his duty as conductor, he ordered some negro tramps to get off of the train, when one of them shot him with a 38-caliber pistol, at short range, the muzzle of the pistol being within a few inches of his body. The ball entered his body in front, at a point about (5) five inches below the left nipple and (1½) one inch and a half outside of the nipple line.

The ball could not be located for several weeks. I saw the patient the first time on September 17th, (nearly a month after the receipt of the injury,) in consultation with his attending physician, Dr. M. M. Butler of Bristol Tennessee. From him I learned that the patient had not had very much fever, the temperature varying from 99° to 101°, pulse from 90 to 100 and not of a sthenic character.

When I saw him, Temperature 102°, very little pain, no cough nor had there been any at any time, no bloody expectoration indicating a wound of the lung; little or no appetite, bowels torpid, breathing somewhat hurried and more pronounced on the right side; patient considerably emaciated.

Physical examination, revealed extensive dullness over a large portion of left chest, amounting to flatness, with absence of normal respiratory sounds and increased percussion resonance, and exaggerated respiratory murmur on right side.

The case not improving, but symptoms of septicæmia becoming more pronounced, I was called to see him again on September 29th with the attending physician.

Physical examination discovered the same condition, somewhat aggravated with the addition of a distinct prominence as large in circumference as the mouth of an ordinary teacup, situated on the back to the left of the spine, corresponding to the eighth and tenth ribs in this region. Taking this as a pointer, we decided to open it.

The patient being under an anæsthetic, a vertical incission was made about three inches in length through, this prominence down to the ribs. On reaching this point there was a gush of very offensive dark fluid amounting to about a quart, which seemed to be a mixture of serum, pus and the debris of disintegrating tissue.

The ball was found just outside of the chest under the deep muscles of the back, having broken a rib (the 10th, I think) about (1½) one and a half inches from the spine in its passage. A portion of the same rib was broken but not detached, about one inch in length, or more, and was hanging loosely in the chest in such a way as to obstruct the opening in the chest wall. This being removed with forceps, the opening was at once filled with a mass of dead tissue forcing its way out.

I began drawing this out, hand over hand, and continued till it quit coming; when I had as much as I could hold in my two hands. It was a stinking mass of dead tissue, consisting I think, of pleura and lung and connective tissue. It was dark, almost black, thickly set with grayish ash-colored spots as large as a wheat grain and larger.

Having removed all this dead tissue, the cavity was washed out with a weak warm solution of carbolic acid, and the wound dressed with iodoform gauze, absorbent cotton and bandage, and the patient quieted with a dose of morphine hypodermically. The temperature steadily declined, the wound being dressed every day and the cavity washed out with warm water, usually with a little carbolic acid added.

The day after the operation we used an enema to evacuate the bowels. The nurse, a negro man, said the water of the injection passed out through the opening in the back. This being doubted, the physician in charge, tested it the next day by injecting into the bowel water colored with Permanganate of Potassium, and he found that the fluid injected into the rectum passed freely and promptly through the opening in the back.

This was an unexpected and perplexing complication.

From this time on, the wound was dressed and washed out every day for some weeks, and for three or four months, fæcal matter passed out through the wound freely, especially when the discharges were thin.

On October 17th I dressed the wound and a mass of fæcal matter as large as a hulled walnut passed out. The patient slowly but steadily improved and is to-day on duty as a railroad conductor, looking well and weighing one hundred and ninety-three pounds, the fistula having entirely closed.

This is an interesting case from the beginning, because—

1st. There were no rational signs pointing to injury of the lung. There was no blood expectorated ; no cough, and at no time was there any of the characteristic sputa of pneumonia.

The case went on for seven weeks, the chest filling with serum and pus, and a large amount of tissue dying within the chest. Yet, aside from the physical signs, and the impaired breathing, there was nothing to indicate the exact nature of the case.

'Tis true, the persistent fever, emaciation, loss of appetite, restlessness and failing strength, indicated something seriously wrong, but it remained for the physical examination to differentiate it.

2d. The ball in its passage had so damaged the diaphragm and the bowel at some point in contact with it as to lead to inflammatory adhesion, followed by sloughing, and resulting in fæcal fistula. And with all this, there was no bloody or mucous discharges from the bowels, and no symptoms indicating injury of the bowel till the water injected into the rectum was seen to pass out through the wound in the back.

In regard to the management of this case, I would say that while no new principle is involved, the result forcibly emphasises the two or three main points in the local treatment of such cases to-day, whether of idiopathic or traumatic origin, viz: cleanliness (or antisepsis, if you please,) with thorough drainage. These given, unless your patient is exhausted from protracted disease, or septicæmia, or some undermining constitutional disease, you are warranted in making a favorable prognosis in most cases.

It is astonishing how promptly convalescence begins after the inauguration of such treatment if not too long delayed ; or if the ordinary "vis medicatrix naturae" is present to assist you. Of course, constitutional treatment, based on general principles, is not to be neglected. The distance from the point of entrance of the ball to the point where it was cut out, around the chest, is 8¾ inches.

